# Supplementary material for: Surface molecular engineering to enable processing of sulfide solid electrolytes in humid ambient air
Source: Nat Commun. 2025 Jan 2;16:213. doi: 10.1038/s41467-024-55634-8 (PMC11696013; doi:10.1038/s41467-024-55634-8)
Supplement: Supplementary file 1 — Supplementary Information [file 41467_2024_55634_MOESM1_ESM.pdf]

Supplementary Information

Surface molecular engineering to enable processing of sulfide electrolytes in humid ambient air

Mengchen Liu[1,=], Jessica J. Hong[1,=], Elias Sebti[2,3,=], Ke Zhou[1,=], Shen Wang[1], Shijie Feng[4], Tyler Pennebaker[2,3], Zeyu Hui[1], Qiushi Miao[4], Ershuang Lu[5], Nimrod Harpak[1], Sicen Yu[4], Jianbin Zhou[1], Jeong Woo Oh[6], Min-Sang Song[6], Jian Luo[1,4], Raphaële J. Clément[2,3]*, Ping Liu[1,4]*

[1]Aiiso Yufeng Li Family Department of Chemical and Nano Engineering, University of California, San Diego, La Jolla, CA, USA

[2]Materials Department, University of California Santa Barbara, Santa Barbara, CA, USA

[3]Materials Research Laboratory, University of California, Santa Barbara, CA, USA

[4]Program of Materials Science, University of California San Diego, La Jolla, CA, USA

[5]Department of Chemistry, University of California, San Diego, La Jolla, CA, USA

[6]LG Energy Solution, Ltd. LG Science Park, Magokjungang 10-ro, Gangseo-gu, Seoul 07796, Republic of South Korea

[=]These authors contributed equally: Mengchen Liu, Jessica J. Hong, Elias Sebti, Ke Zhou

E-mail: rclement@ucsb.edu; piliu@eng.ucsd.edu

This file includes:

Supplementary Fig. 1 to 28

Supplementary Table 1

Supplementary Note 1 to 4

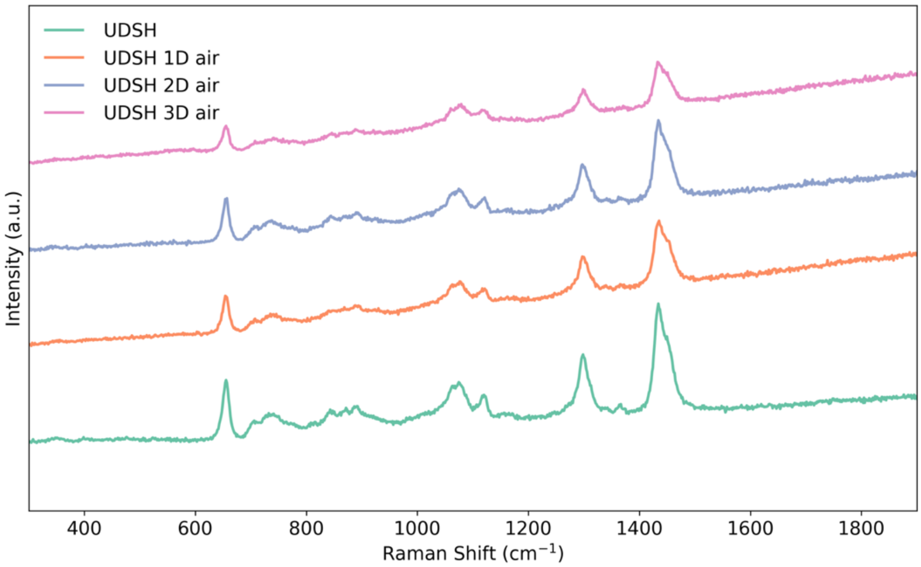

Supplementary Fig. 1 Raman spectroscopy of a pure 1-undecanethiol (UDSH) solution in the pristine state and after 1, 2 and 3 days of exposure to air with 33% RH (labeled as UDSH 1D air, UDSH 2D air and UDSH 3D air). Source data are provided as a Source Data file.

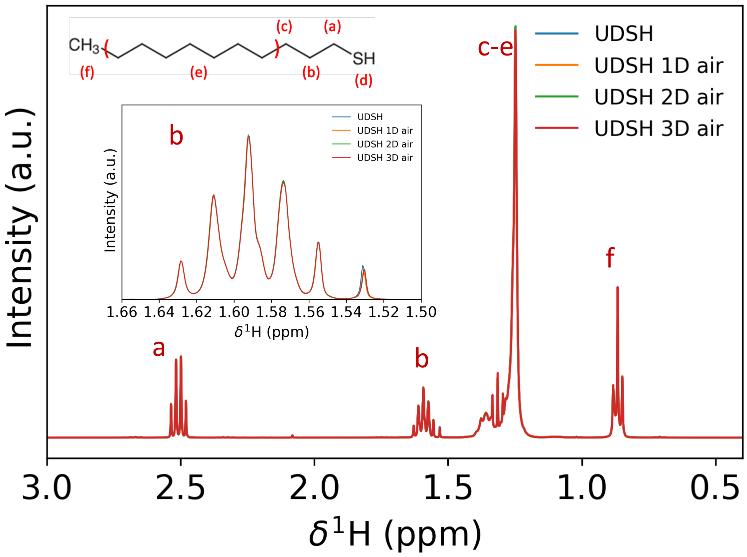

Supplementary Fig. 2 ^{1}H liquid NMR spectra of a pure UDSH solution in the pristine state and after 1, 2 and 3 days of exposure to air with 33% RH (labeled as UDSH 1D air, UDSH 2D air and UDSH 3D air). Source data are provided as a Source Data file.

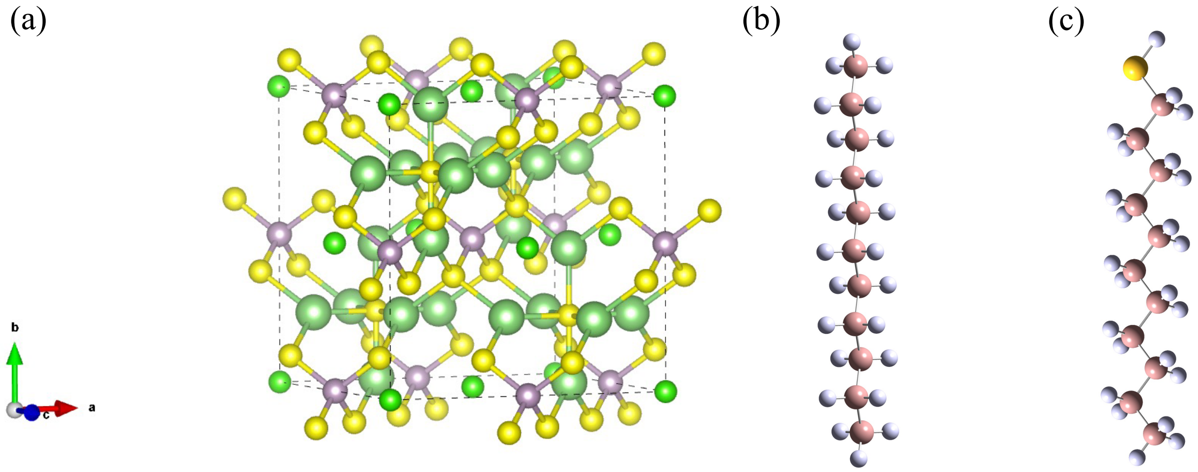

Supplementary Fig. 3 Optimized atomic structures of the three systems of interest in this work. (a) Li_6PS_5Cl (LPSC), (b) undecane(UDCH), and (c) 1-undecanethiol (UDSH). The six types of atoms are represented by colored spheres as follows: Li (light green), P (light purple), S (yellow), Cl (bright green), C (light Orange), and H (Silver).

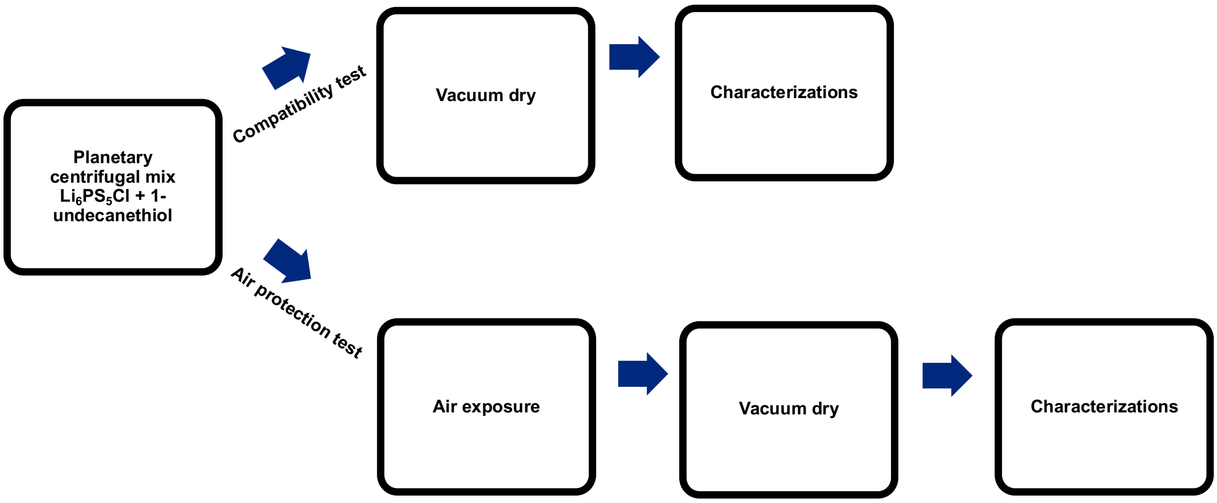

Supplementary Fig. 4 Synthesis processes of surface modified electrolyte. The process includes the following sequence: planetary centrifugal mixing of LPSC and UDSH with Thinky, subject to air exposure or without air exposure, 80 °C vacuum drying for 2 hours to remove free thiol molecules, and structural characterization.

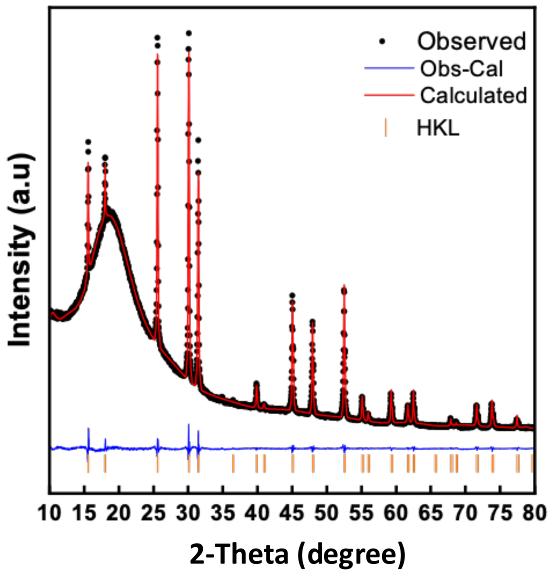

Supplementary Fig. 5 Rietveld refinement of the XRD pattern obtained on an UDSH@LPSC without air exposure. Source data are provided as a Source Data file.

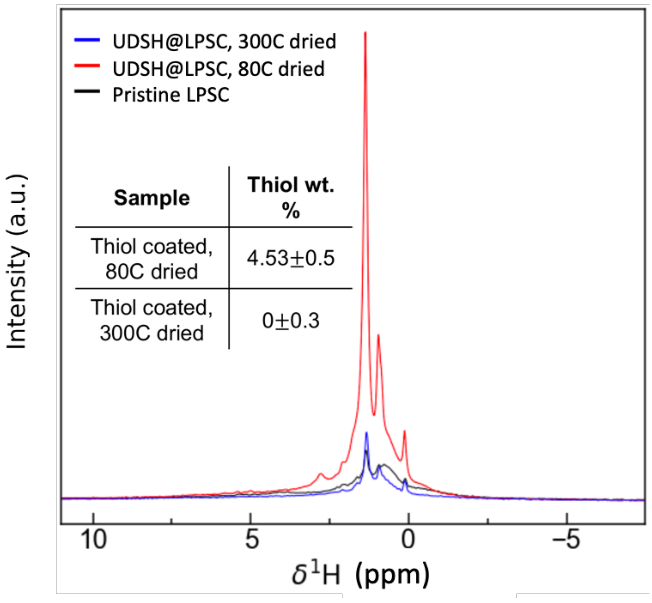

Supplementary Fig. 6 ¹H spin echo NMR spectra collected on pristine LPSC (black color), UDSH@LPSC after different drying conditions: vacuum drying at 80°C for 2 hours (red color); vacuum drying at 80°C for 2 hours with an additional heat treatment at 300°C for 3 hours (blue color). Source data are provided as a Source Data file.

Supplementary Note 1: UDSH residue quantification

All spectra in **Supplementary Fig. 6** were obtained at 18.8 T and at a 20 kHz MAS speed. Spectra were scaled to have the same number of scans and total sample mass. All other measurement conditions were identical and recycle delays of 5*T1 were used. The inset table reports the weight percent of UDSH in the dried UDSH@LPSC samples, determined from fits and integration of the ^{1}H NMR signals in the spectra. We note that, while signals from the UDSH surface modifier dominate the 1H spectrum collected on the UDSH@LPSC, UDSH residues only represent a small mass fraction of the sample, as only a very small ^{1}H signal arises from the LPSC phase. Some of the 1H signal intrinsic to the LPSC (see spectrum obtained on the pristine LPSC) is lost during the heat treatment at 300 °C. In thiol quantification, the 1H signal from the pristine material is subtracted from the total signal intensity to isolate contributions from the UDSH. Doing this on the sample that was heated at 300 °C results in a negative signal intensity, indicating that some of the protons in the pristine LPSC are evolved out of the sample during the 300 °C heating.

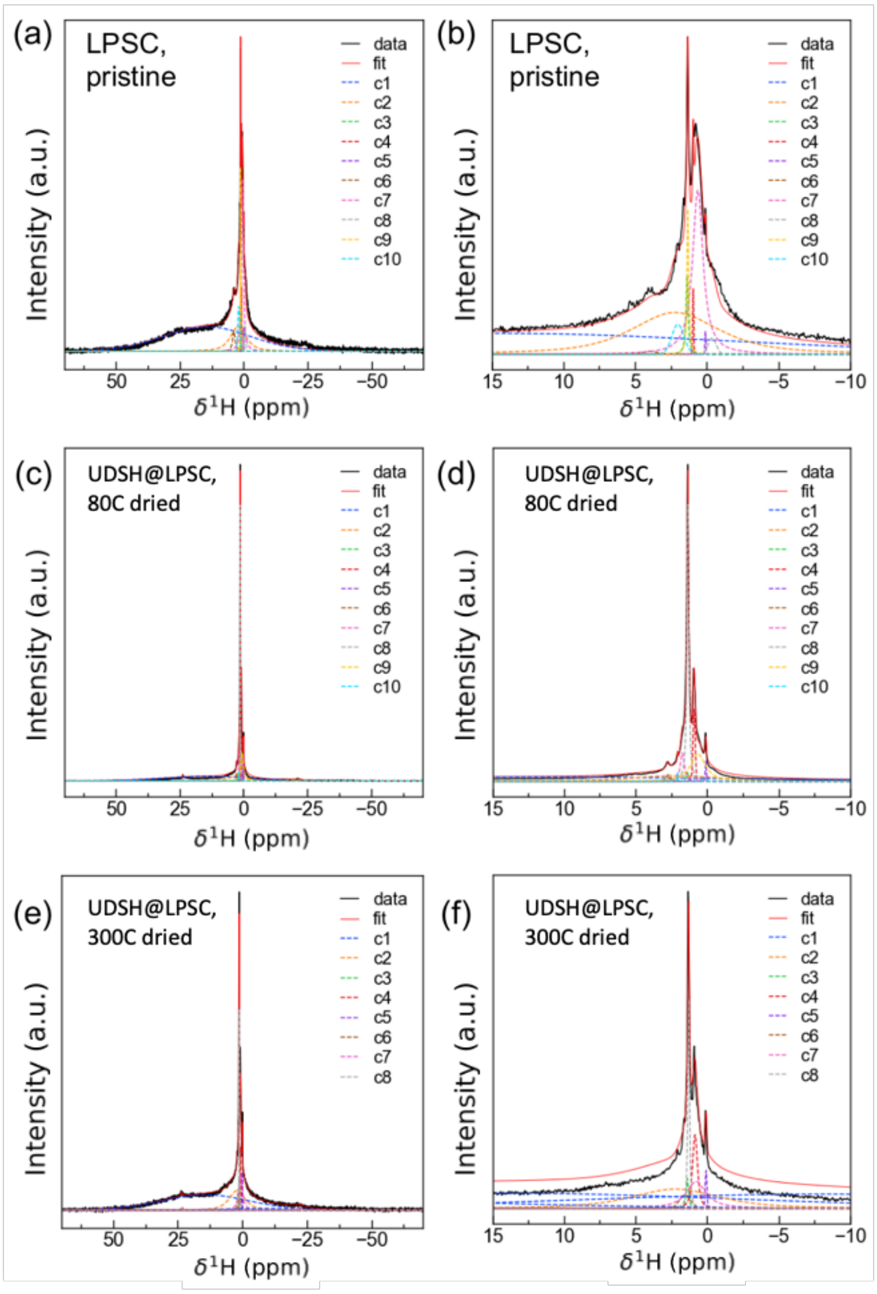

Supplementary Fig. 7 Representative fits from 1H quantification of UDSH residue in UDSH@LPSC samples. ^{1}H spin echo NMR spectra collected on (a-b) pristine LPSC, (c-d) UDSH@LPSC vacuum drying at 80°C for 2 hours, (e-f) UDSH@LPSC samples vacuum drying at 80°C for 2 hours with an additional heat treatment at 300°C for 3 hours. All spectra were obtained at 18.8 T and at a 20 kHz MAS speed. T2* measurements were conducted to fit the decay of each component and compensate for lost signal during the 50 µs echo delay. Source data are provided as a Source Data file.

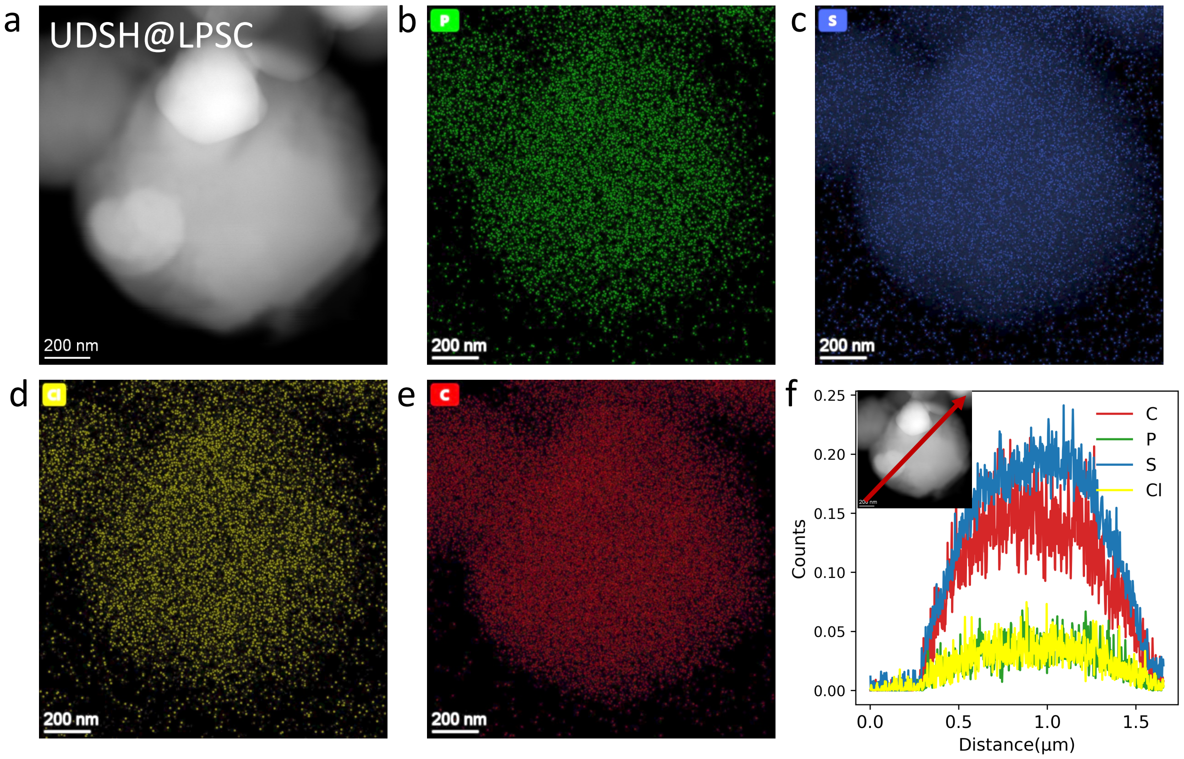

Supplementary Fig. **8** Characterization of UDSH@LPSC by Cryo-TEM. (a) HAADF image. (b)-(e) corresponding elemental maps for phosphorus, sulfur, chlorine, and carbon. (f) elemental distribution from line scan. Source data are provided as a Source Data file.

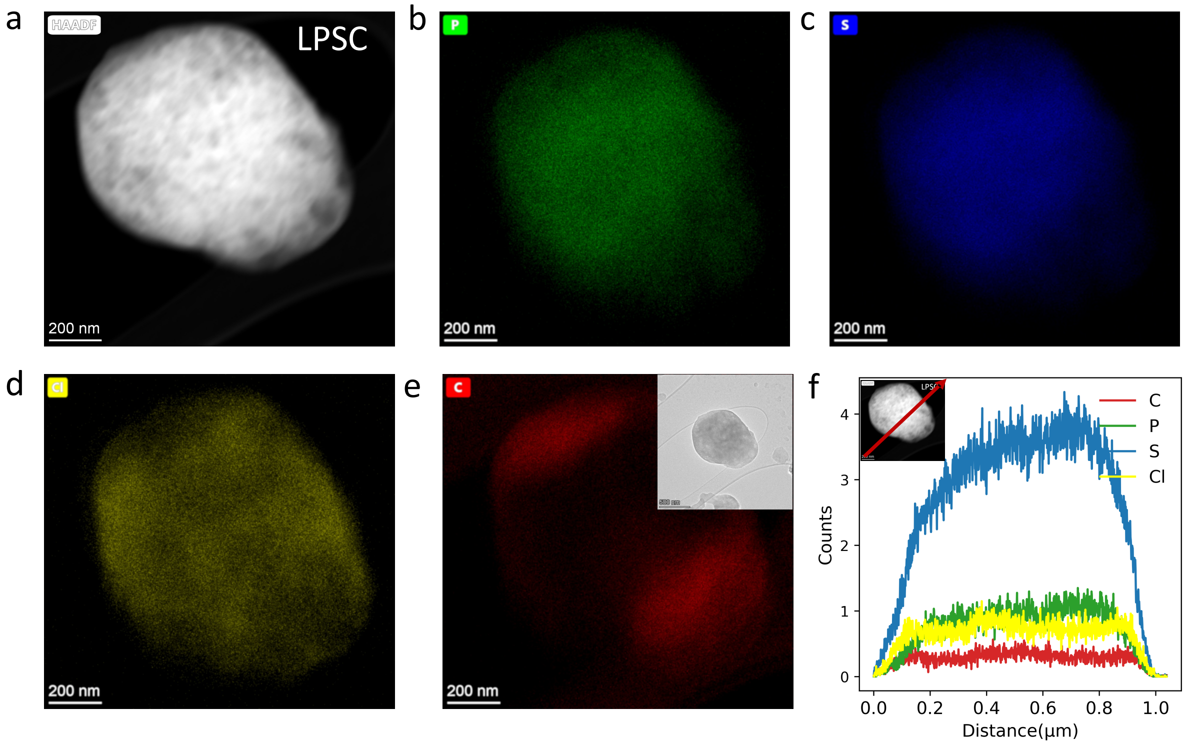

Supplementary Fig. 9 Characterization of LPSC by Cryo-TEM. (a) HAADF image. (b)-(e) corresponding elemental maps for phosphorus, sulfur, chlorine, and carbon. (f) elemental distribution from line scan. Source data are provided as a Source Data file.

**Element**	**Mass Fraction %**	
	**LPSC**	**UDSH@LPSC**
**C**	**1.07**	**9.31**
**P**	**17.84**	**12.39**
**S**	**82.23**	**63.31**
**Cl**	**8.86**	**14.99**

Supplementary Table 1 Elemental mass fraction of LPSC and UDSH@LPSC measured by Cryo-TEM EDX.

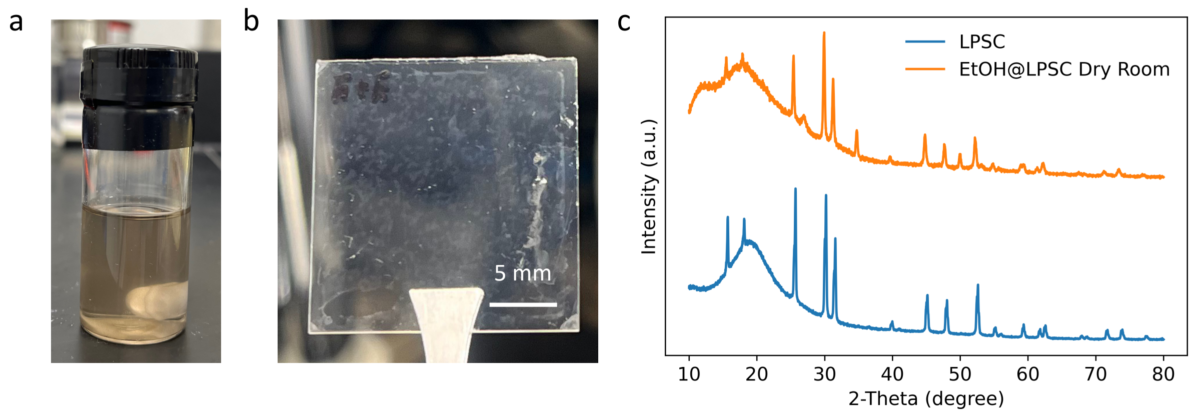

Supplementary Fig. 10 Nanometer scale thin film LPSC preparation. (a) LPSC dissolved in anhydrous ethanol with a concentration of 0.1 wt%, labeled as EtOH@LPSC. (b) Spin coated EtOH@LPSC on glass in the dry room with a dew point of -40°C. (c) XRD pattern of EtOH@LPSC after heat treatment at 180°C as compared to that of pristine LPSC. Source data are provided as a Source Data file.

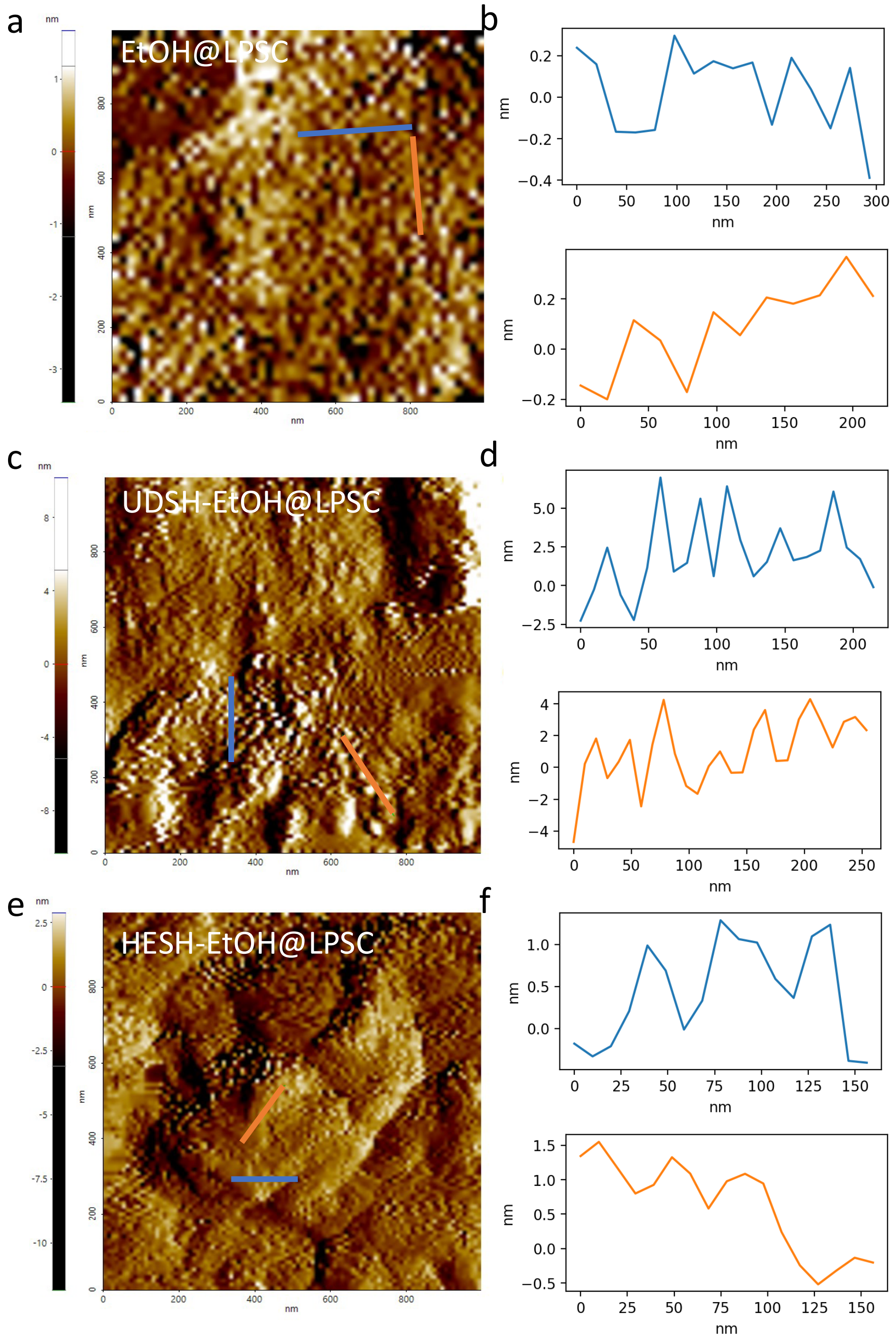

Supplementary Fig. 11 Characterization of structural configuration of UDSH and HESH on LPSC by AFM. Morphological (height) images and line profiles of EtOH@LPSC (a-b), UDSH-EtOH@LPSC (c-d), and HESH-EtOH@LPSC (e-f). Source data are provided as a Source Data file.

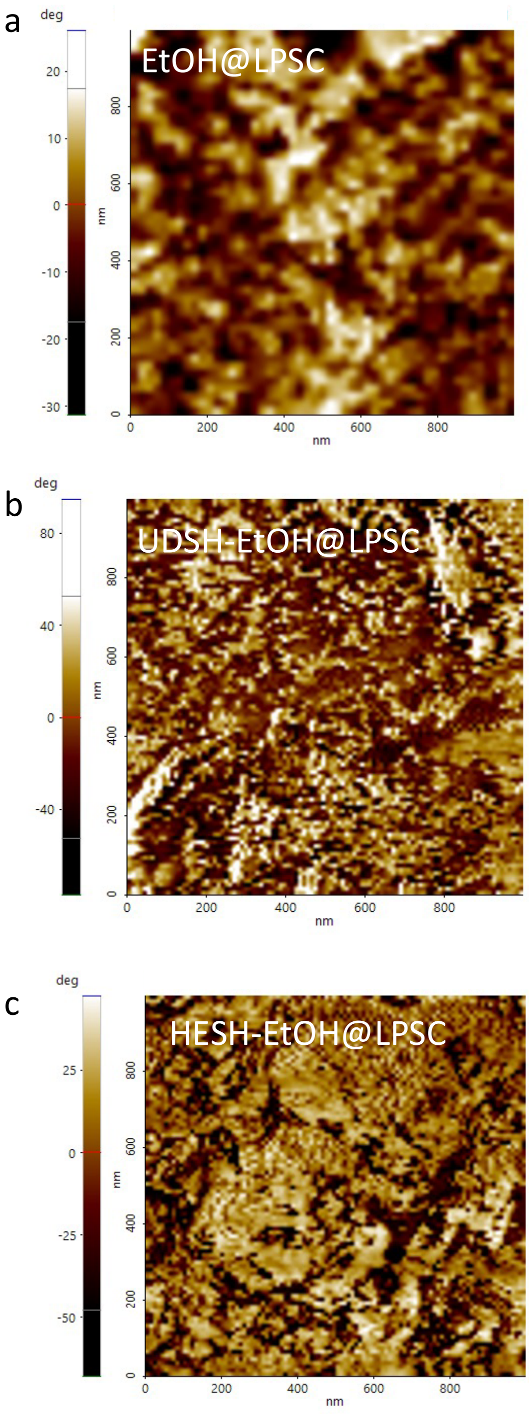

Supplementary Fig. 12 Characterization of phase configuration of UDSH and HESH on LPSC by AFM. Phase images of thin film EtOH@LPSC (a), UDSH-EtOH@LPSC (b), and HESH-EtOH@LPSC (c).

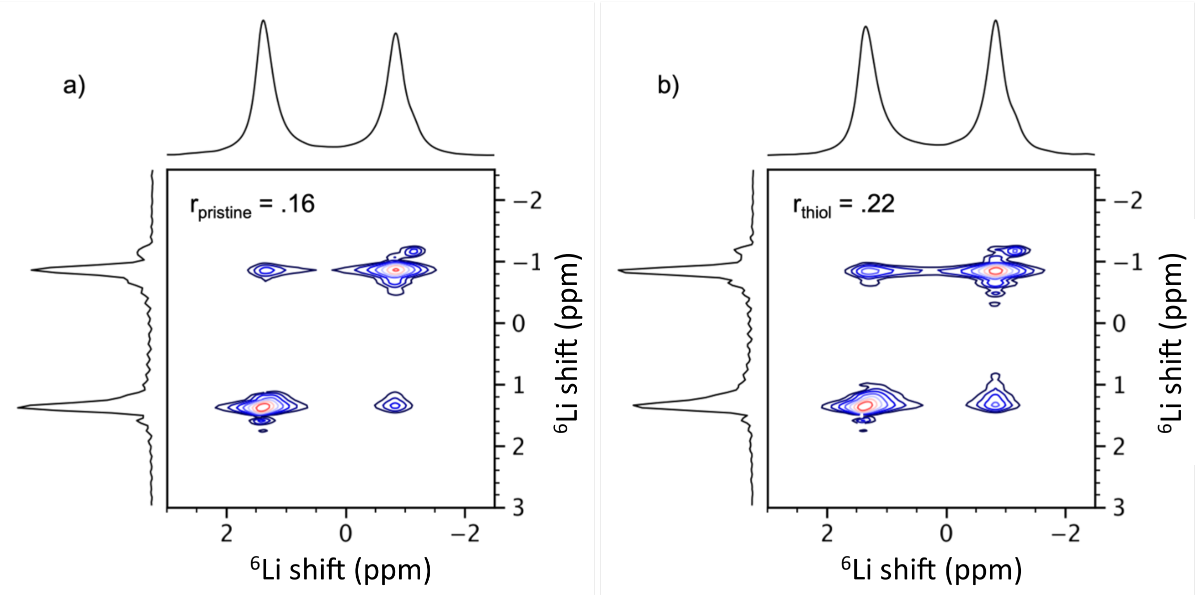

Supplementary Fig. 13. The role of the UDSH molecule in influencing interfacial ion transport. ^{6}Li 2D EXSY spectra collected on a) LZC + Pristine LPSC, and b) LZC + UDSH@LPSC powder mixtures to demonstrate that Li can diffuse through the thiol-coating layer. Oscillations in the second dimension are caused by truncation of the free induction decay curve due to experimental limitations. The spectra were acquired at 18.8 T and at 30 kHz MAS using a mixing time τ_{mix} of 100 ms. Source data are provided as a Source Data file.

Supplementary Note 2: Discussion on UDSH's effect on ionic conductivity through 2D NMR

To demonstrate that the UDSH coating layer does not preclude Li-ion transport in and out of the LPSC particles, we compared the rate of Li chemical exchange between LPSC and the Li_2ZrCl_6 (LZC) solid electrolyte, and between UDSH@LPSC and LZC using ^{6}Li 2D exchange spectroscopy (EXSY). The 2D EXSY spectra collected on powder mixtures of LPSC and LZC, and UDSH@LPSC and LZC, are presented in **Supplementary Fig. 13**. Here UDSH coated LPSC is mixed with another solid ion conductor, Li_2ZrCl_6 (LZC). The diagonal peaks represent lithium nuclei which remained in the same environment (same solid state electrolyte phase) during the given mixing time ($\tau_{m\mathrm{ix}}$). The cross-peaks, on the other hand, correspond to the Li species that exchanged environments over the course of the experiment, i.e., diffused through the LPSC-LZC or UDSH@LPSC-LZC interface. In agreement with prior studies, the ^{6}Li NMR signal arising from the LPSC phase has a shift of ~1.4 ppm and the ^{6}Li NMR signal arising from the LZC phase appears at approximately -0.8 ppm [1,2]. 2D gaussian lineshapes were fit to the diagonal and cross peaks to extract their intensities and determine the ratio between the total cross-peak intensity and the total diagonal intensity:

$$r = \frac{I_{LPSC,LZC} + I_{LZC,LPSC}}{I_{LPSC,LPSC} + I_{LZC,LZC}}$$

This quantity provides a direct measurement of the fraction of Li species exchanged between the two environments (solid state electrolytes) of interest over the course of the experiment. Because both measurements were taken at a mixing time of 100 ms and all environments involved have similar T_1 relaxations times of 4.2 to 7.3 s $\gg \tau_{m\mathrm{ix}}$, the intensity ratios can be directly compared to evaluate exchange in the LPSC and in the UDSH@LPSC systems. The cross-peak intensity ratio is 0.16 for pristine LPSC with LZC and 0.22 for UDSH@LPSC with LZC, indicating that a greater proportion of Li species have exchanged between the two solid electrolytes in the latter system over the 100 ms mixing period. Hence, the UDSH coating does

not prevent diffusion in and out of the LPSC solid electrolyte particles, and instead facilitates such transport.

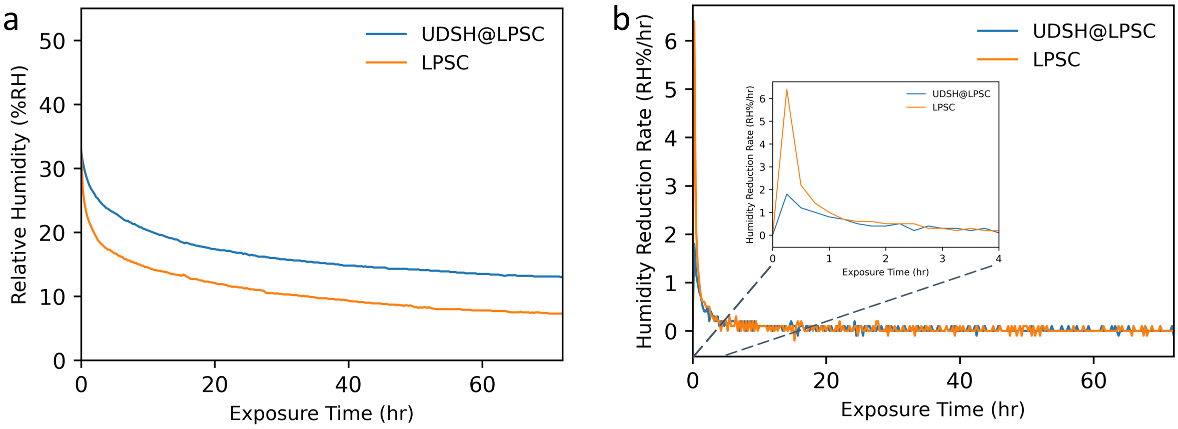

Supplementary Fig. 14 Water uptake of LPSC and UDSH@LPSC samples exposed to air with 33% RH. (a) The water uptake of LPSC and UDSH@LPSC samples was quantified through the drop in relative humidity inside the vessel as a function of time; (b) The rate of water uptake, i.e., the first order derivative of curves in (a). The lower rate of water uptake in UDSH@LPSC indicate its better moisture stability than pure LPSC. Source data are provided as a Source Data file.

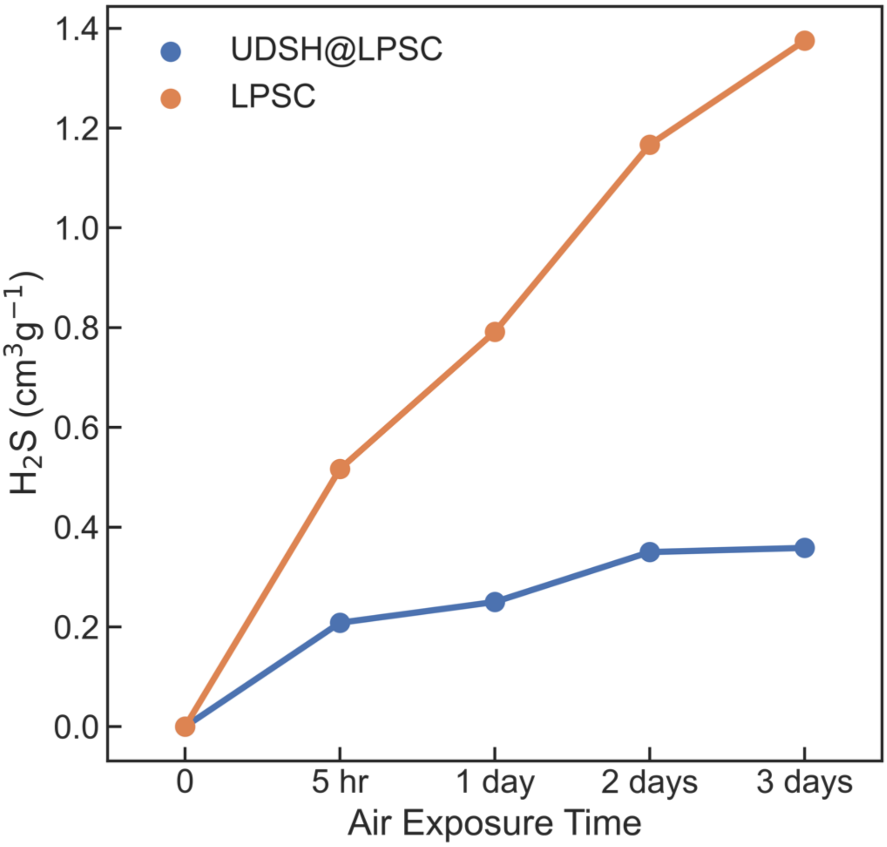

Supplementary Fig. 15 Evolution of H_2S generation during LPSC and UDSH@LPSC exposure in 33%RH ambient air. Source data are provided as a Source Data file.

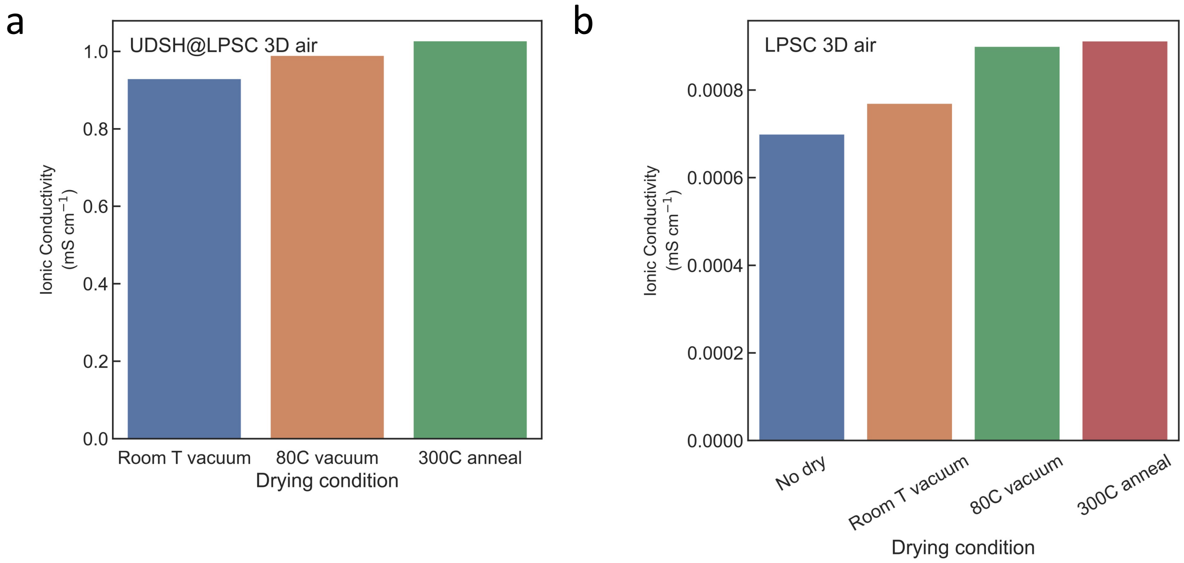

Supplementary Fig. 16 Negligible effect on the ionic conductivity of electrolytes after vacuum drying at 80°C for 2 hours. (a) The ionic conductivity of UDSH@LPSC 3D air after different drying condition: vacuum drying at room temperature (blue color); vacuum drying at 80°C for 2 hours (orange color); vacuum drying at 80°C for 2 hours followed by at 300°C for 3 hours in Ar (green color). (b) The ionic conductivity of LPSC 3D air sample after different drying condition: without any drying treatment (blue color); vacuum drying at room temperature (orange color); vacuum drying at 80°C for 2 hours (green color); vacuum drying at 80°C for 2 hours followed by at 300°C for 3 hours in Ar (red color). Source data are provided as a Source Data file.

Supplementary Note 3: Discussion on the effect of 80ºC thermal treatment on ion conductivity

In our work, a treatment at 80°C for two hours primarily aims to eliminate excess UDSH for sample pelletizing and ionic conductivity measurement, rather than to restore its structure and conductivity. Without drying treatment, the UDSH modified LPSC samples have the texture of a wet paste due to the presence of liquid UDSH, which can be expelled under pressure during the EIS test, complicating the test's reliability. Further, we emphasize that after the exposure test in moisture, both the uncoated LPSC and UDSH@LPSC samples undergo the same vacuum drying process at 80°C for 2 hours prior to property characterization. This ensures a fair comparison between UDSH@LPSC and LPSC.

To more directly examine the potential impact of 80°C vacuum drying, we also conducted the following experiments. As shown in **Supplementary Fig. 16a,** UDSH@LPSC 3D air samples were vacuum dried at room temperature to remove excess UDSH without applying any heat treatment. The ionic conductivity was measured to be 0.93 mS cm^{-1}, comparable to the value of 0.99 mS cm^{-1} for the sample vacuum dried at 80°C. Furthermore, when the UDSH@LPSC 3D air samples were heat-treated at 300°C for 3 hours, the ionic conductivity remained nearly unchanged at 1.03 mS cm^{-1}, again confirming the negligible impact of thermal treatment. In **Supplementary Fig. 16b,** the ionic conductivity of LPSC 3D air sample under different drying conditions were measured. Without any drying treatment, the ionic conductivity was measured to be 0.0007 mS cm^{-1}, comparable to the value of 0.00077 and 0.0009 mS cm^{-1} for sample vacuum dried at room temperature or vacuum dried at 80°C for 2 hours. Furthermore, when the LPSC 3D air samples were heat-treated at 300°C for 3 hours, the ionic conductivity remained nearly unchanged at 0.00091 mS cm^{-1}, again indicating that the vacuum drying process had negligible effects. These ionic conductivity measurements collectively confirm that the 80°C 2-hour heat treatment has no discernible effect on conductivity.

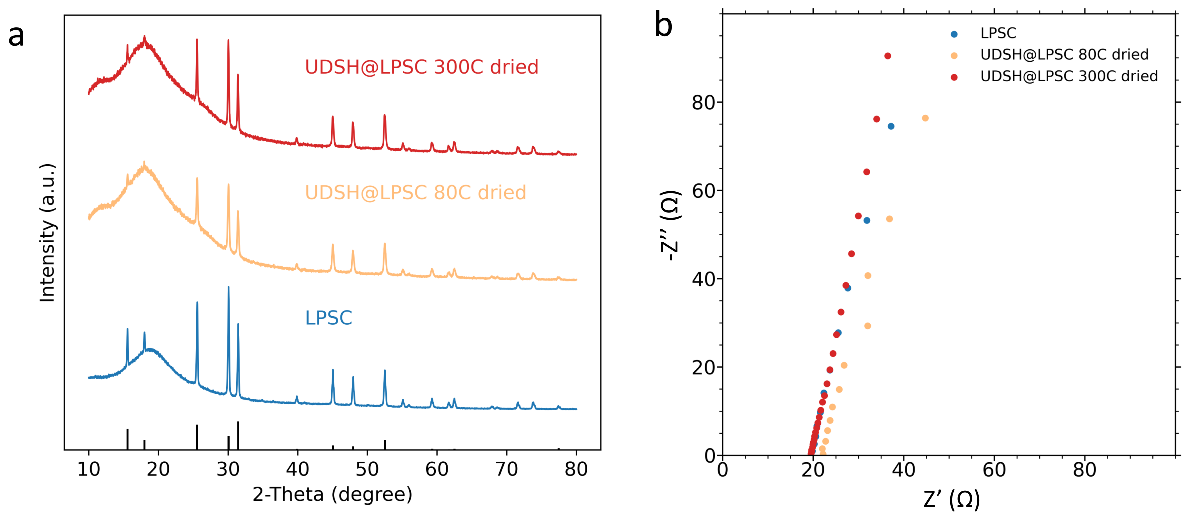

Supplementary Fig. 17 Complete removal of all UDSH residues through a 300°C heating process. (a) XRD patterns and (b) Nyquist plots of pellets sandwiched between two titanium rods made of LPSC, UDSH@LPSC vacuum dried at 80°C for 2 hours and UDSH@LPSC vacuum dried at 80°C for 2 hours followed by at 300°C heat for 3 hours, respectively. Source data are provided as a Source Data file.

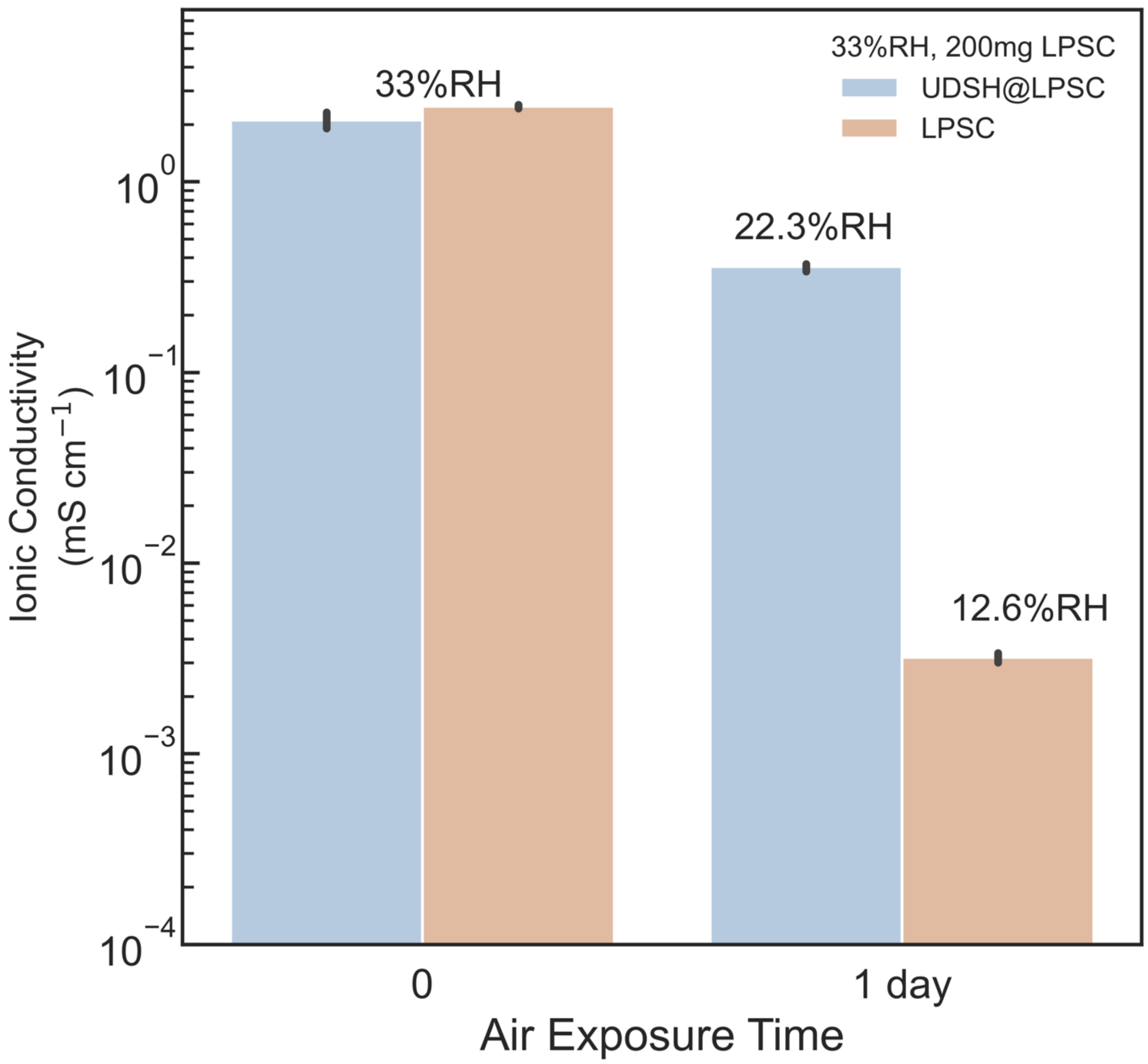

Supplementary Fig. 18 Ionic conductivity of 200mg LPSC and UDSH@LPSC (200mg of LPSC and 40mg of UDSH) as a function of air exposure time and relative humidity, based on the average of 3 independent measurements, errors correspond to one standard deviation. Data are presented as mean values +/- SD. Source data are provided as a Source Data file.

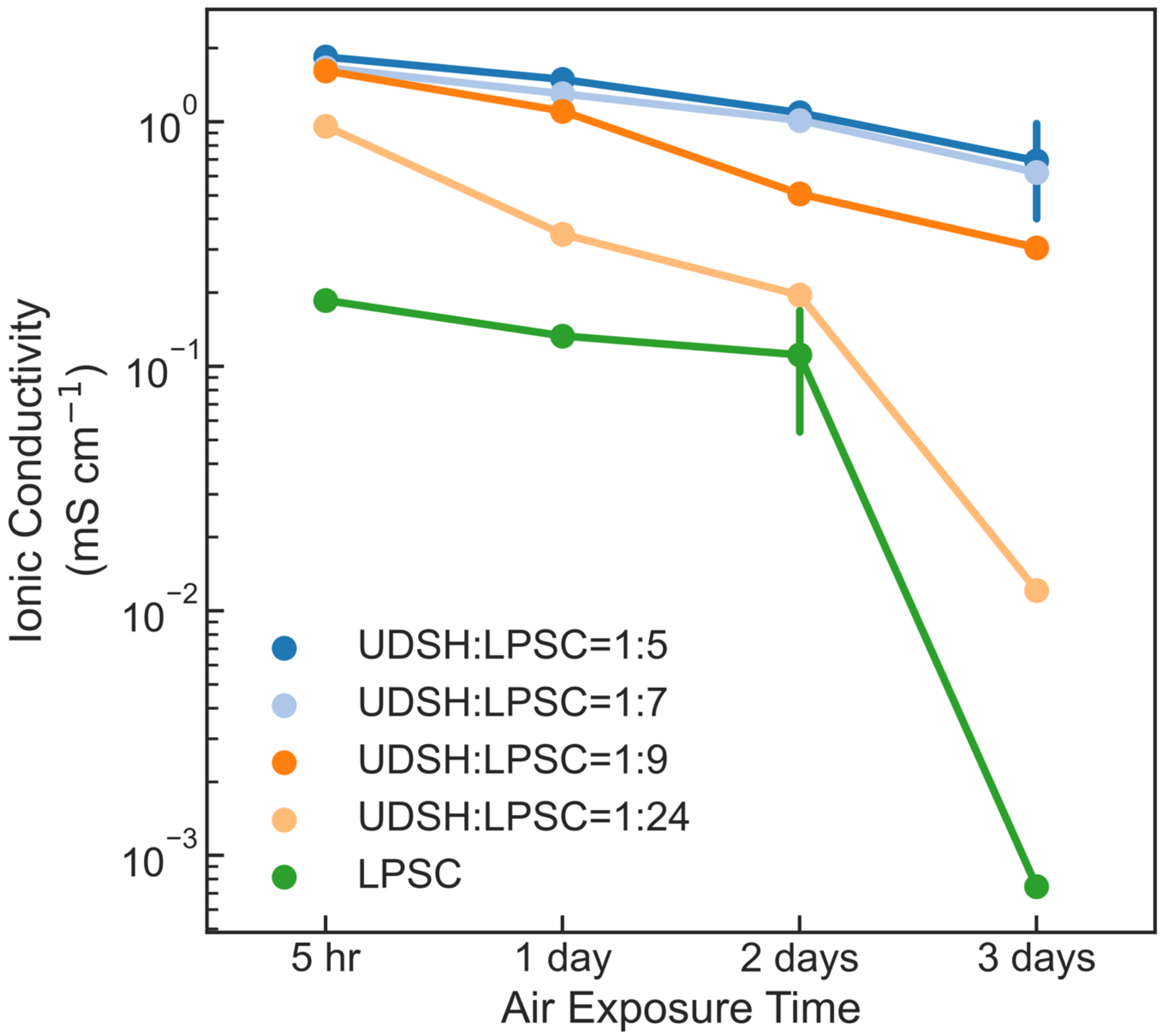

Supplementary Fig. 19 Effect of UDSH ratio on LPSC stability. Ionic conductivity of LPSC and UDSH@LPSC with different weight ratios of UDSH:LPSC=1:5, 1:7, 1:9, 1:24 as a function of exposure time in 33% RH air. Based on the average of 2 independent measurements (errors correspond to one standard deviation). Data are presented as mean values +/- SD. Source data are provided as a Source Data file.

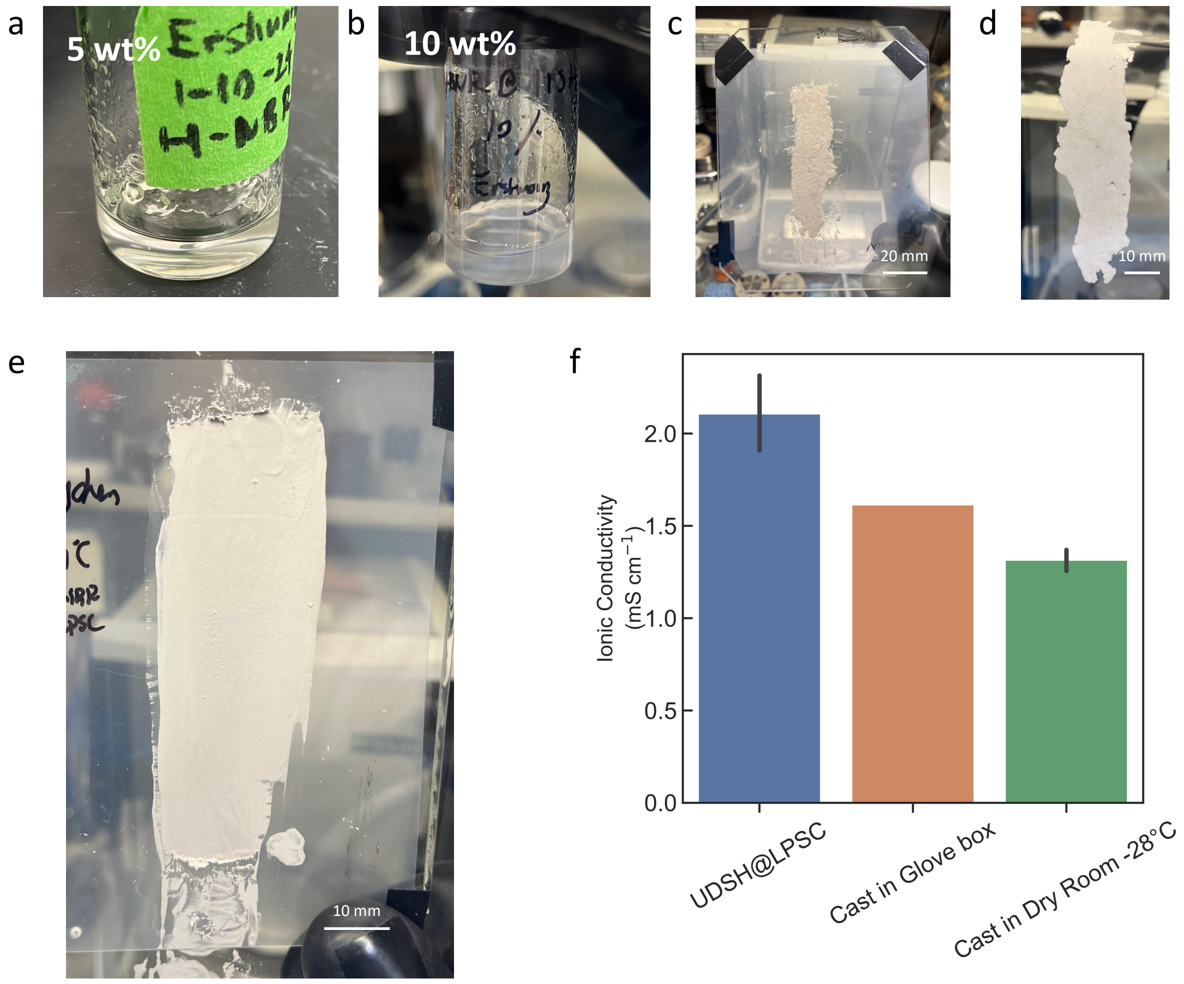

Supplementary Fig. 20 Use of UDSH as solvent for a polymer binder to process SSE films even in a less controlled environment, due to its protection effect. Dissolution of (a) 5wt% (b) 10wt% HNBR in UDSH at room temperature. The casting of a film from a LPSC/5wt%HNBR/UDSH paste within (c)-(d) Ar filled glove box and (e) dry room with dew point of -28ºC; (f) Ionic conductivity of UDSH@LPSC and the two films obtained in (d) and (e). Ionic conductivity of UDSH@LPSC and cast in dry room shown in (f), based on the average of 3 independent measurements (errors correspond to one standard deviation). Data are presented as mean values +/- SD. Source data are provided as a Source Data file.

Supplementary Note 4: Discussion on SSE film processing with UDSH

UDSH can dissolve the common binder and solvent for SSE. Hydrogenated nitrile butadiene rubber (HNBR) can dissolve in UDSH with a solubility of 10 wt% at room temperature (**Supplementary Fig. 20a-b).** UDSH is also miscible with toluene, attaining a weight percentage of 20 wt%. We fabricated LPSC electrolyte films by casting from a paste made of LPSC powder dispersed in a 5 wt% HNBR solution in UDSH. The films were processed within an Ar filled glove box (**Supplementary Fig. 20c-d**) or in a less controlled environment (dry room with a dew point of -28° C) (**Supplementary Fig. 20e**). After vacuum dried at 80°C, the resultant films show conductivities of 1.6 mS cm^{-1} and 1.3 mS cm^{-1}, respectively (**Supplementary Fig. 20f**). These findings show that UDSH has great potential to be a multifunctional agent, enabling processing of SSE films while protecting it from moisture-induced degradation.

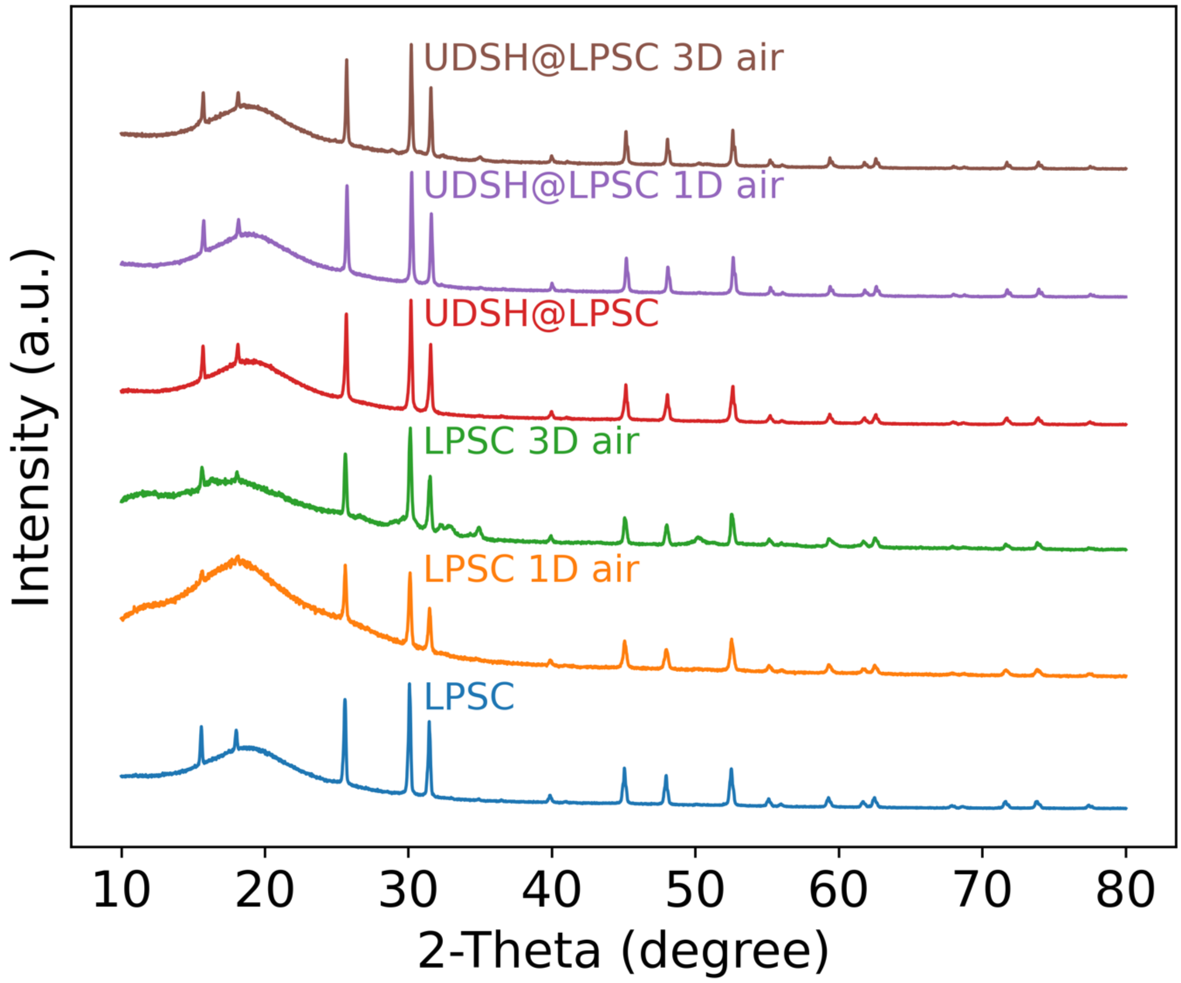

Supplementary Fig. 21 XRD patterns collected on LPSC and UDSH@LPSC samples exposed to 33%RH air from 1 day to 3 days. Source data are provided as a Source Data file.

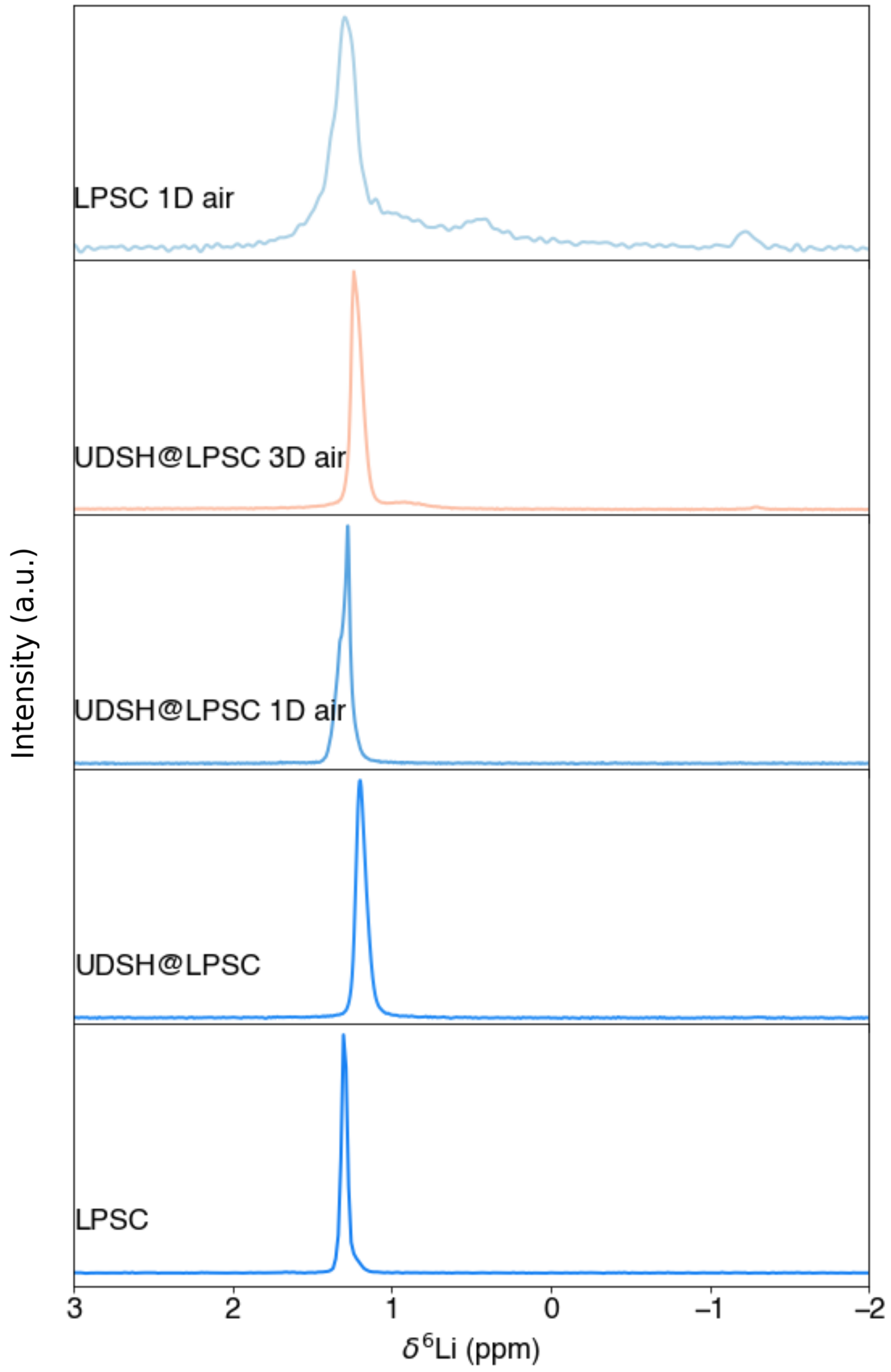

Supplementary Fig. 22 ^{6}Li spin echo ssNMR spectra collected on LPSC and UDSH@LPSC samples exposed to air from 0 to 3 days with 33% RH. Spectra were obtained at 18.8 T under 20 kHz of magic angle spinning. Source data are provided as a Source Data file.

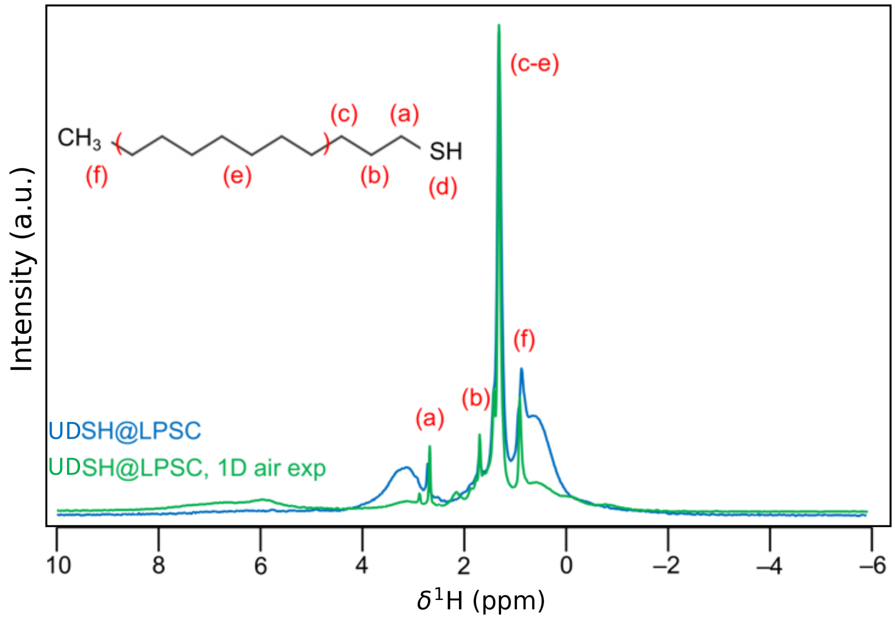

Supplementary Fig. 23 ^{1}H spin echo ssNMR spectra collected on UDSH@LPSC and UDSH@LPSC exposed to air with 33% RH for 1 day. Spectra were obtained at 18.8 T under 10 kHz of magic angle spinning. Source data are provided as a Source Data file.

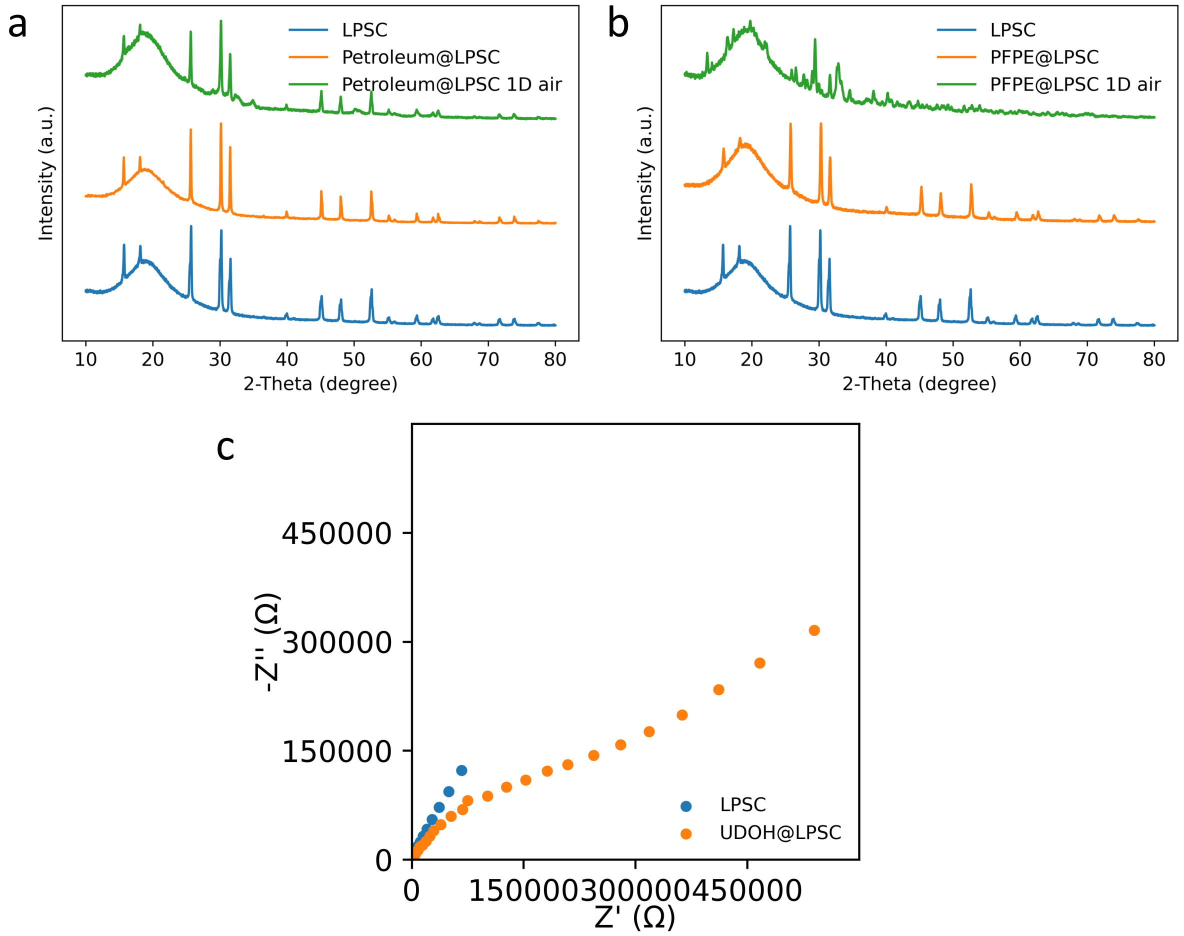

Supplementary Fig. 24: Air protection effect of hydrophobic materials with different functional groups on LPSC. XRD patterns collected on (a) LPSC protected by petroleum wax and (b) PFPE oil before and after 1 day of air exposure. (c) Nyquist plot of LPSC and undecanol coated LPSC (UDOH@LPSC) tested under room temperature without air exposure. Source data are provided as a Source Data file.

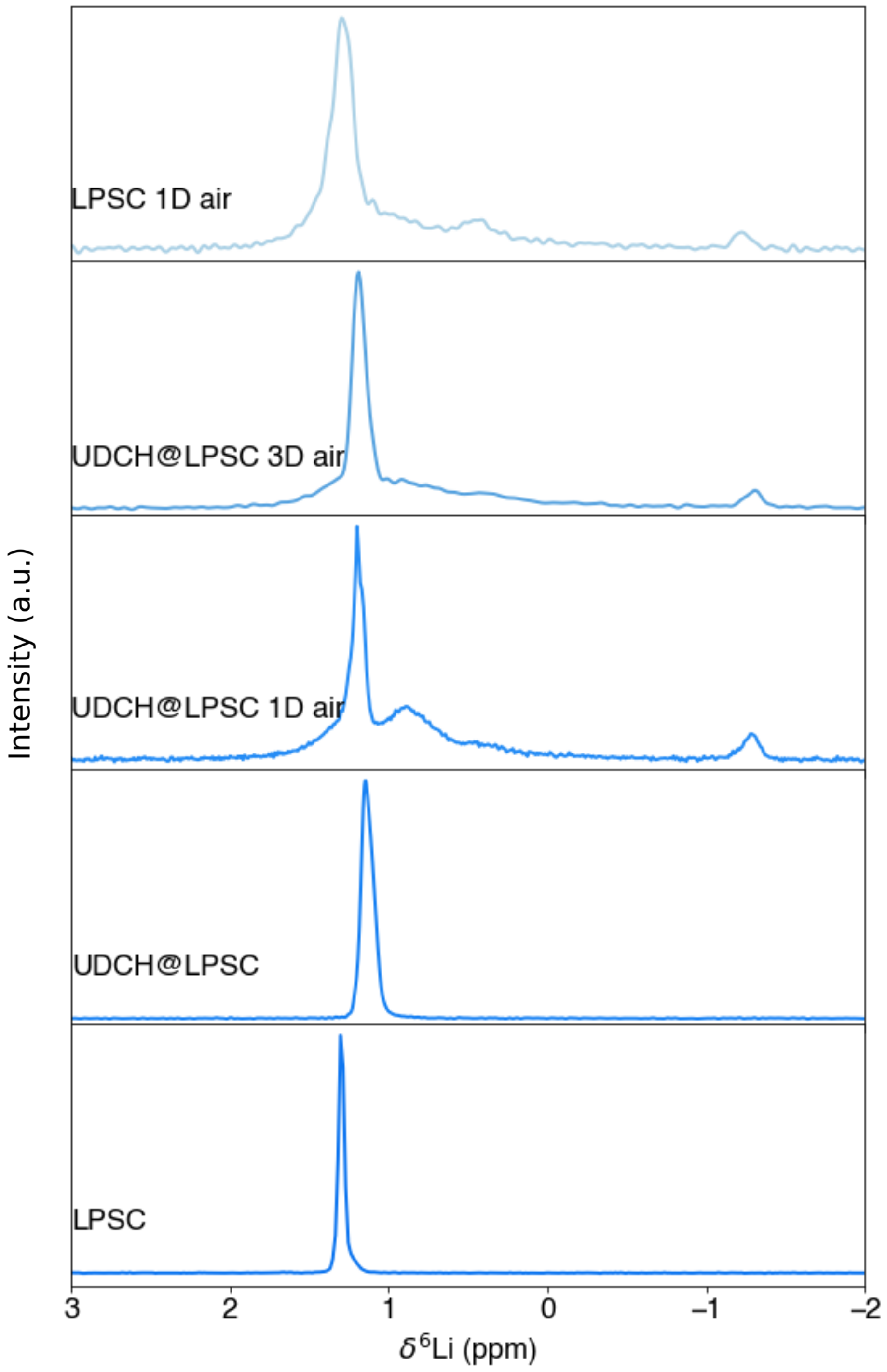

Supplementary Fig. 25 ^{6}Li spin echo ssNMR spectra of LPSC and UDCH@LPSC from 0 day to 3 days air exposure with 33%RH humidity. All spectra were obtained at 18.8 T under a MAS speed of 20 kHz. Source data are provided as a Source Data file.

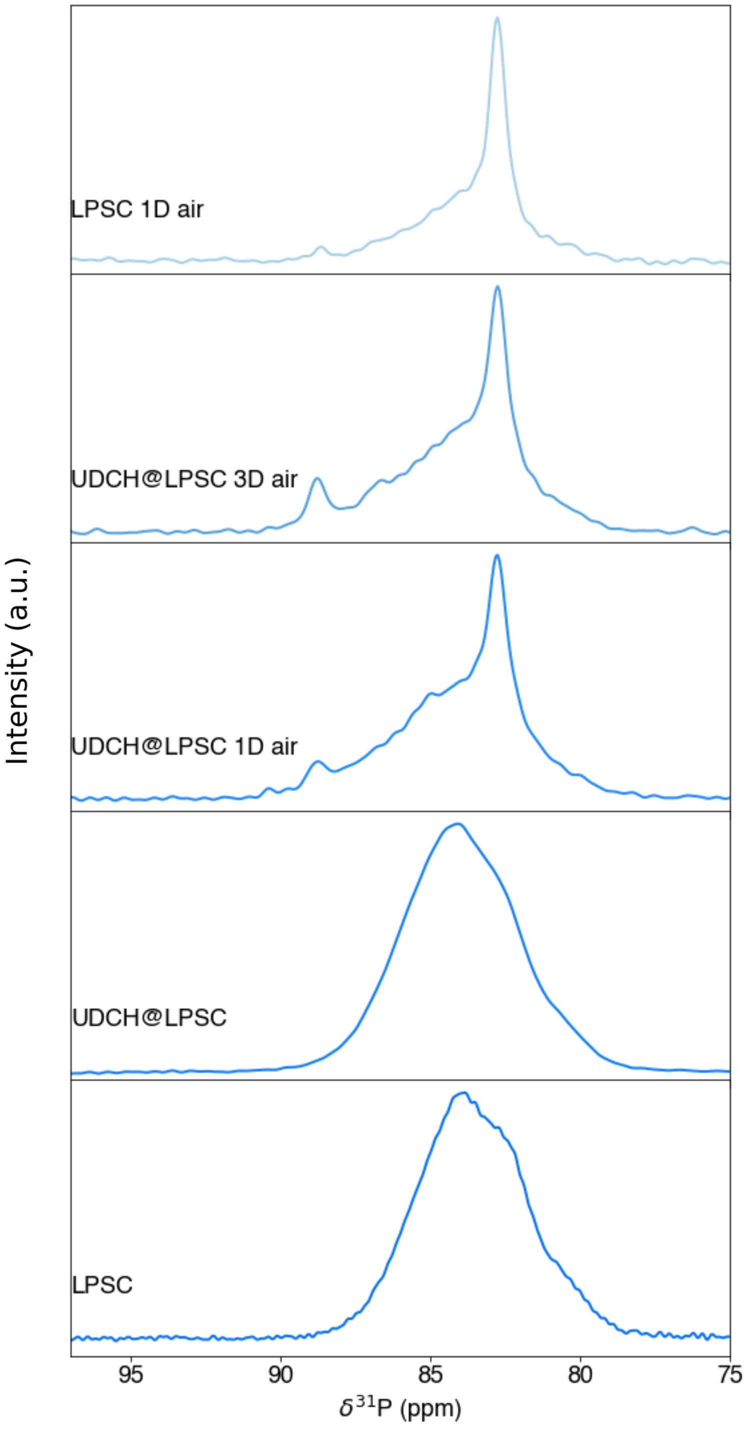

Supplementary Fig. 26 ^{31}P spin echo ss-NMR spectra of LPSC and UDCH@LPSC from 0 day to 3 days air exposure with 33% RH humidity. All spectra were obtained at 18.8 T under a MAS speed of 60 kHz. Source data are provided as a Source Data file.

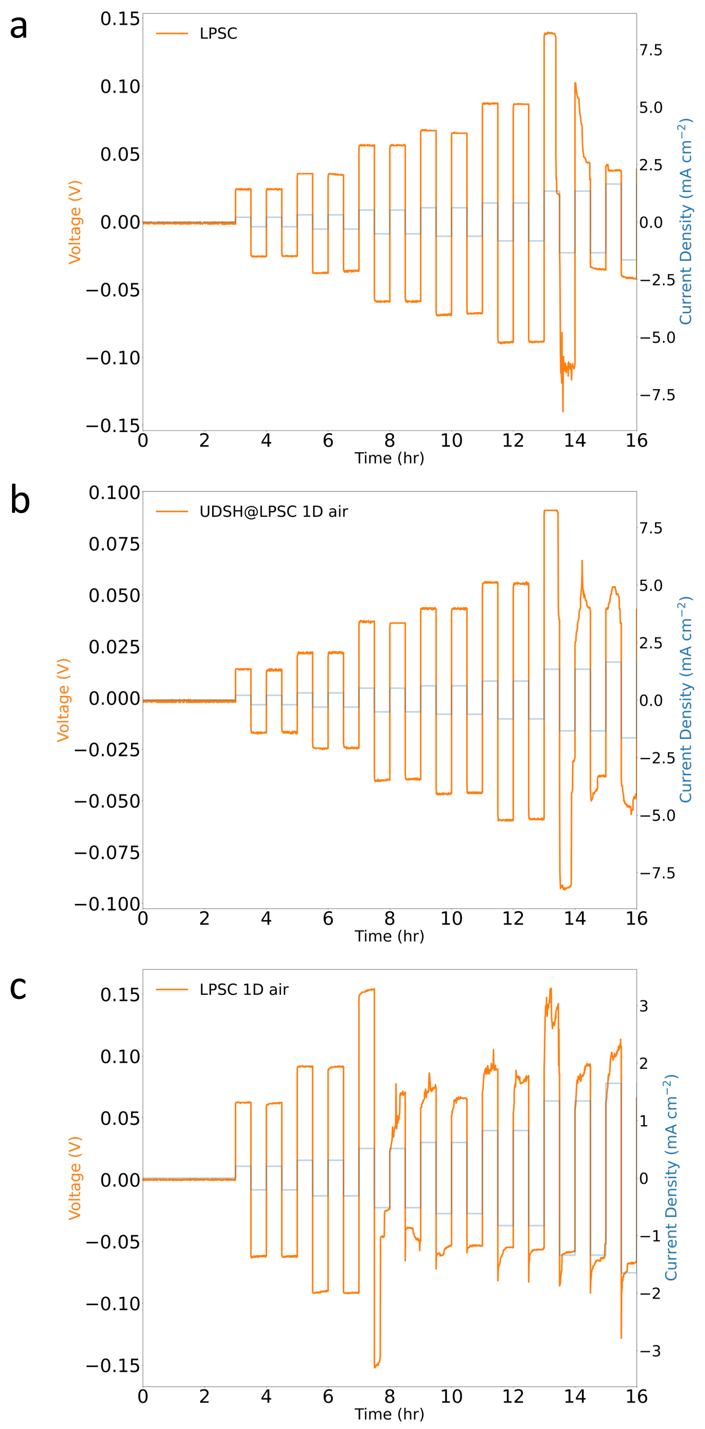

Supplementary Fig. 27 Symmetric cell performance. Critical current density of Li||Li symmetric cell with electrolyte layer made of (a) LPSC, (b) UDSH@LPSC 1D air and (c) LPSC 1D air under room temperature. Source data are provided as a Source Data file.

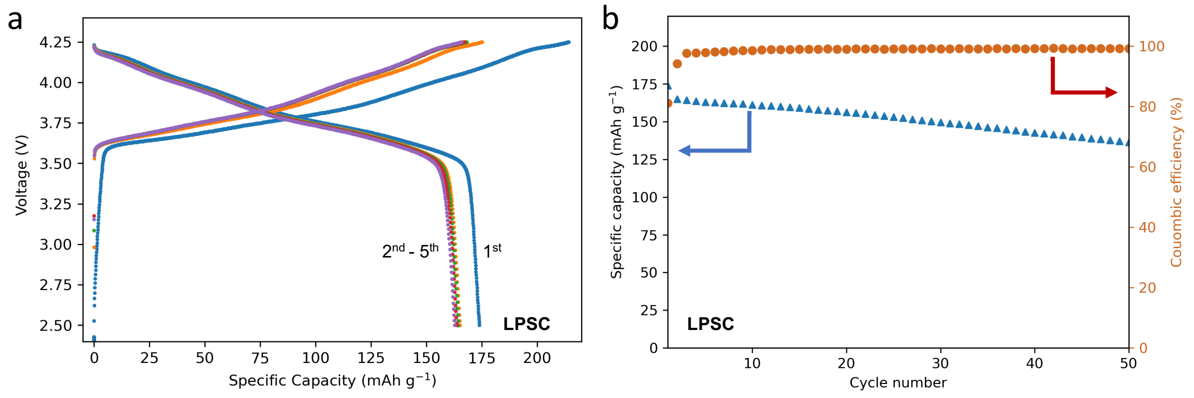

Supplementary Fig. 28 Full cell cycling performance. (a) Voltage profiles of an $Li_{0.5}In$|LPSC|NCM811 composite cell over the first 5 cycles at 60 °C and at 0.15 mA cm^{-2}. (b) 50 cycle capacity retention for the cell shown in (a). Source data are provided as a Source Data file.

Supplementary References:

1. Kwak, H. et al. Boosting the interfacial superionic conduction of halide solid electrolytes for all-solid-state batteries. Nat Commun 14, (2023).
2. Ganapathy, S., Yu, C., Van Eck, E. R. H. & Wagemaker, M. Peeking across Grain Boundaries in a Solid-State Ionic Conductor. ACS Energy Lett 4, 1092–1097 (2019).
